# Live Intravital Imaging of Cellular Trafficking in the Cardiac Microvasculature—Beating the Odds

**DOI:** 10.3389/fimmu.2019.02782

**Published:** 2019-11-26

**Authors:** Dean Philip John Kavanagh, Neena Kalia

**Affiliations:** Institute of Cardiovascular Sciences, College of Medical and Dental Sciences, University of Birmingham, Birmingham, United Kingdom

**Keywords:** cardiac imaging, motion artifact detection, motion artifact removal, intravital imaging, microcirculation, ischaemia and reperfusion injury, cardiac microcirculation

## Abstract

Although mortality rates from cardiovascular disease in the developed world are falling, the prevalence of cardiovascular disease (CVD) is not. Each year, the number of people either being diagnosed as suffering with CVD or undergoing a surgical procedure related to it, such as percutaneous coronary intervention, continues to increase. In order to ensure that we can effectively manage these diseases in the future, it is critical that we fully understand their basic physiology and their underlying causative factors. Over recent years, the important role of the cardiac microcirculation in both acute and chronic disorders of the heart has become clear. The recruitment of inflammatory cells into the cardiac microcirculation and their subsequent activation may contribute significantly to tissue damage, adverse remodeling, and poor outcomes during recovery. However, our basic understanding of the cardiac microcirculation is hampered by an historic inability to image the microvessels of the beating heart—something we have been able to achieve in other organs for over 100 years. This stems from a couple of clear and obvious difficulties related to imaging the heart—firstly, it has significant inherent contractile motion and is affected considerably by the movement of lungs. Secondly, it is located in an anatomically challenging position for microscopy. However, recent microscopic and technological developments have allowed us to overcome some of these challenges and to begin to answer some of the basic outstanding questions in cardiac microvascular physiology, particularly in relation to inflammatory cell recruitment. In this review, we will discuss some of the historic work that took place in the latter part of last century toward cardiac intravital, before moving onto the advanced work that has been performed since. This work, which has utilized technology such as spinning-disk confocal and multiphoton microscopy, has—along with some significant advancements in algorithms and software—unlocked our ability to image the “business end” of the cardiac vascular tree. This review will provide an overview of these techniques, as well as some practical pointers toward software and other tools that may be useful for other researchers who are considering utilizing this technique themselves.

## Heart Disease and Inflammation

In the United Kingdom, more than a quarter of all deaths are attributable to cardiovascular disease (CVD) and around 7.4 million people currently live with a heart or circulatory disease (1). While mortality is decreasing in developed countries, the number of people who are living with cardiovascular conditions is increasing; the number of patients in the UK who underwent percutaneous coronary interventions (PCI) in 2013 was seven times the number two decades earlier (2). Although there has been significant progress in the reduction of mortality rates from CVD, it is still the cause of death for almost 170,000 people every year– accounting for almost 28% of all mortality in the UK (3). In order to fully ensure that we can treat this disease properly in the next decade, it is critical that we fully understand the mechanisms that underlie this condition.

One of the key players in both acute and chronic heart conditions is inflammation. The role of inflammation in heart disease has become clearer in recent years. For many years, heart disease was considered to be a problem of hemodynamics, resulting simply from blockage or narrowing of blood vessels (4). It subsequently became apparent that a haemodynamic theory alone could not explain the manner in which heart failure (HF) progressed. This led those in the field at the time to suggest additional underlying mechanisms contributing to the development of HF. The “cytokine hypothesis,” proposed in the early 1990s, suggested that it was in fact the release of cytokines during heart disease that drove the progression and pathology of this disorder (5). Since then, and particularly over the last 10 years, we have become increasingly aware of the importance of inflammation and the role leukocytes play in the progression of heart disease. Whether inflammation *causes* heart disease or vice-versa is a hotly debated topic, and probably varies on a patient-by-patient basis. Although circulating pro-inflammatory cytokine levels are raised in patients with HF and positively correlate with disease severity (6), this does not necessarily indicate that the inflammation is the predominant causative factor. Much of our understanding of the role of inflammation in heart disease is derived from animal models. For instance, animals over expressing TNFα have high levels of inflammatory infiltrate in their hearts and develop dilated cardiomyopathy; these mice have an exceptionally high mortality rate (~25% at 6 months) (7). Interleukin-23p19^−/−^ mice, who have an interleukin-23 deficiency (a cytokine important in the differentiation of CD4+ cells), show significantly increased inflammation, impaired scar formation, and adverse remodeling after MI (8). Consistent with this, using anti-inflammatory approaches has been shown to be beneficial in animal models of heart disease; administration of a TNFα antagonist attenuates the development of myocardial inflammation, fibrosis, and subsequent cell death in a model of streptozotocin-induced diabetic cardiomyopathy (9).

Exposure of cardiac endothelial cells to pro-inflammatory cytokines leads to the upregulation of adhesion molecules (10), which in turn, leads to the recruitment of inflammatory effector cells. These effector cells, which include neutrophils, monocytes, macrophages, and lymphocytes, can induce apoptotic, and phenotypic changes in cardiac endothelial cells via the release of cytokines, reactive oxygen species, or the engagement of counter-ligands on the endothelial cell surface (4). Endothelial cell phenotypic changes in this manner are not trivial—TGF-β and Ang-II can induce an endothelial-to-mesenchymal transition, shifting endothelial cells toward a fibroblast phenotype and leading to the development of cardiac fibrosis (11). It is clear, however, that some degree of inflammatory cell infiltrate is required for normal repair functions to take place following an ischaemic event or during the development of a chronic cardiac pathophysiological disease state (12). It is widely accepted and understood that inflammatory cells are often required for the resolution and repair of injured tissues. Indeed, that is also the case with the heart—for instance, monocytes/macrophages are essential for normal physiological healing of the heart following MI (13). However, what is important from a therapeutic point of view is that we are able to ensure that the inflammatory response to an insult is appropriately measured and does not overwhelm the local tissue environment. In particular, the main goal is to protect the local microvascular environment as it is within the microcirculation where the inflammation that is causative for HF is thought to predominantly occur (14).

## The Importance of the Coronary Microcirculation—the “Business End” of the Vascular Tree

The potential role of the coronary microcirculation in pathologies of the heart has been known for some time. In 1967, Likoff et al. described a set of 15 patients who they considered to have coronary heart disease, but with patent coronary arteries (15). With remarkable foresight, Likoff et al. suggested that the coronary syndrome exhibited in these patients resulted from “abnormalities in the microcirculation” and that “an oxygen-diffusion impairment … at these levels could be responsible for the symptoms and signs of apparent myocardial ischemia” (15). Over the subsequent 50 years, our understanding of the importance of the microvasculature in coronary pathophysiology has improved significantly and we are now acutely aware of the importance of coronary microvascular dysfunction (CMVD). In 2013, CMVD was implicated as a primary causative factor for heart failure with preserved ejection fraction (HFpEF), shifting the causative emphasis away from left ventricular afterload excess (14). This suggestion is consistent with the idea that smaller vascular components are critical in determining the vascular resistance of the heart; ~55% of the total vascular resistance in the heart originates in cardiac microvessels (16).

Understanding how the microvessels of the heart operate in health and disease is critical. The heart is supplied by two major coronary arteries—the left and right—which both originate from the early part of the ascending aorta. These arteries branch into smaller vessels which supply distinct anatomical regions of the heart. This branching initially occurs at the epicardium, before continuing into the myocardium where the vessels begin to form a tree-like network (17). Finally, in contrast with a number of other tissue beds, these vessels develop into a non-tree like network with hairpin loops, T-, Y-, and H-shaped junctions (18). Interestingly, as a result of these connections, capillaries which are directly adjacent to each other may have completely opposite and counter-current flow profiles (19). Our understanding of microvascular perfusion in the cardiac microvasculature is further complicated by the contractile activity of the heart. Intramyocardial microvessels are subject to rhythmic compression during systole (20) with diameters decreasing by up to 20% (21). Furthermore, blood which enters the coronary arterioles during diastole can be squeezed out during systole, in some cases generating retrograde flow (22). Although one would assume similar physical forces to be applicable in the capillaries, the extent to which retrograde flow occurs in the terminal end of the microcirculation is currently unclear. This highly contorted structural design, in conjunction with the complexities of contraction-dependent retrograde and counter-current flow, makes *in vitro* or *in silico* modeling of the coronary microvasculature challenging. The ability to image the coronary microvasculature *in situ* is a critical step in helping us to understand the nature of pathophysiology that occurs in this bed.

Unfortunately, research investigation of the microcirculation in patients is difficult. Techniques such as positron emission tomography (PET) have allowed the identification of some cardiac specific haemodynamic parameters, such as myocardial blood flow (MBF; mL/min/g) and coronary flow reserve (CFR; MBF near maximal coronary vasodilation to basal MBF) (23). Single photon emission computed tomography (SPECT), magnetic resonance imaging (MRI), and ultrasound offer some structural information, but often without sufficient resolution to identify “true” microvasculature (24). While these are useful surrogate markers for coronary vascular dynamics, none of them are—in practical terms—suitable for clinicals or researchers to directly visualize the coronary microcirculation in patients (25). Compounding this situation, imaging the coronary microcirculation *in vivo* has also been considered, at least for the vast majority of the last 50 years, particularly challenging. This has led to some researchers referring to the cardiac microcirculation as a research “black box” (25). Not only is the heart in an anatomically challenging location in respect of imaging, the physiological motion inherent to the organ makes identification of the microvasculature difficult. Recent work has identified the size of the challenge facing scientists in regard cardiac motion. Of all of the major organs used for intravital microscopy, the heart has one of the highest maximal displacements under normal physiological conditions (up to 19.9 mm/s) (26); worse still, this displacement more than doubles when animals are on ventilation (47.8 mm/s) (26), an essential component during surgery for intravital imaging of the heart. As a result of this movement, without stabilization techniques, resolution at a single cell level is impossible—something which intravitalists have been able to achieve in other vascular beds for over a hundred years (27). This has led to the of significant deficits in our knowledge related to the coronary microcirculation and how this relates to cardiac diseases and pathophysiology (25). While many other tissue beds are readily available for intravital microscopy (28–33), the unique nature of the coronary microcirculation limits our ability to use these organs as surrogates for the study of this important vascular network.

## Imaging the Coronary Microcirculation—the Early Studies

Experimental imaging studies of the microcirculation began to gather pace during the mid-to-late part of the 1900s. These studies could be categorized into three main categories: (1) those aimed at identifying the layout of the coronary microvascular network under normal physiological conditions and under conditions of hypoxia; (2) those examining the effect of cardiac contraction on the coronary microcirculation; and (3) studies seeking to understand the effects of myocardial ischemia reperfusion injury on the coronary microcirculation. In perhaps the first feasible example of cardiac intravital, Martini and Honig (34) performed microscopy on the beating heart of ventilated rats. In this preparation, the heart was exposed via an incision in the chest wall, covered with a glass slip, and illuminated via a point-strobe light driven into a ring condenser. While this model generated useable images, it did not control for movement. So poor was movement control, that the authors required “~100–150 feet of film to provide 30–50 focused frames” (34).

Subsequent studies began to address this issue of stabilization. These studies all generally shared the same technical setup which was indicative of the time—images were captured using camera-based systems, with the tissue illuminated either by either epi- or trans-illumination. All of these preparations (and indeed, their modern counterparts) had to deal with two main physiological processes which induce movement—cardiac contraction and pulmonary inflation. In one of the first imaging studies of the beating canine heart, Tillmanns et al. used a number of adaptations to overcome the inherent difficulties with imaging the beating organ (35). Firstly, the authors considered epi-illumination—while easy to achieve—to be inefficient. As the ventricular wall is too thick for transillumination, light needed to be transmitted toward the microscope objective from a point *within* the heart muscle. This method of transillumination was achieved using what, even some 45 years later, might still be considered an elegant approach. Tillmanns et al. used a 20G needle, engineered to contain a quartz rod (which is conducive to light) with a mirrored end, with the terminal end modified to reflect light at 45°. Insertion of this under the superficial layer of the myocardium would lead to the transmission of light upwards and through the ventricular wall, making capture by light microscopy viable (35). In addition, to ensure that the microscope remained in focus with the surface of the beating heart, Tillmanns et al. designed what they termed a “focus keeper”—a floating, counterbalanced system that allowed the microscope objective to move in vertical synchronicity with the beating heart. This ensured that the objective always retained an identical z-distance from the tissue surface regardless of the movement of the heart itself. While controlling for movement in the XZ plane, it should be noted that this mechanism did not specifically include mechanisms that compensated for lateral movement; rather, the application of the device with some downward force was expected to hold the heart in its lateral position (35). This group went on to further validate this method, publishing evidence of vascular patency and perfusion dynamics through imaging of the administration of FITC-conjugated dextran (19).

Following on from these initial studies, intravital microscopy of the heart continued to develop, with particular focus around the development of methods to improve tissue stability and resultant image quality. Originally, Nellis et al. attempted to mechanically (and relatively aggressively) fix a segment of the beating heart around a specific point, allowing them to image this area free from movement artifacts (36). However, they quickly found that aggressive mechanical fixation significantly impeded microvascular perfusion (36). Consequently, they slightly adapted their existing method to be more conservative in its approach to constraint. In this study, the authors designed two imaging techniques for the beating rabbit heart; (1) a free-motion imaging technique, where transillumination came from a light source underneath the tissue surface (thus, inside the ventricle) but with no mechanism to constrain the imaging site with relation to the light; (2) a fixed-position imaging technique, where the trans/epi-illumination also included a means for holding the tissue in positional synchronicity with the light source (37). In addition, the authors were one of the first groups to utilize electrocardiographic (ECG) gating to help minimize motion artifacts. However, rather than being used to trigger imaging, the ECG was linked to a stroboscopic light source. Using the QRS complex as a reference point, Nellis and colleagues were able to trigger rapid and short-lived (15–25 μs) illumination at the same point in the cardiac cycle (38). While the heart is moving relatively free at all times, only illuminating the surface at a particular point in the cardiac cycle gives a perception of stillness. Furthermore, slightly staggering the triggering delay from the QRS complex allowed the authors to give an impression of the heart moving in slow motion.

Pulmonary inflation proved a much more challenging issue to deal with; while the heart could be either physically constrained or movement compensated for, preventing the lungs from inflating is clearly an exceptionally difficult task in the context of most surgical setups. After many years, (39) identified high-frequency jet ventilation (HFJV) as a potential technique which could be used to mitigate some of this movement. HFJV is a relatively non-conventional mechanism of ventilation which is thought to be favorable in cohorts who are at risk of chronic lung injury (40). It maintains effective gas exchange, yet effectively reduces respiratory motion to almost nil, by working at very small tidal volumes and high respiratory rates (41). By applying this methodology to cardiac intravital imaging, the authors were able to ventilate the lungs, but with minimal physical displacement of the heart due to pulmonary movement. Cardiac displacement was further dampened by the positioning of 22G needles entering at the interventricular groove and exiting from the left ventricle (39) and tissue movement negated by the use of stroboscopic illumination. In a further technological advance, the authors designed a semi-automated electromagnetically controlled micromanipulator—termed the “*Wobbler*.” This device could be registered manually with the location of the vasculature of interest at various points during the cardiac cycle. Once this information was known, an attached computer was able to move the micromanipulation arm in synchronicity with the vessel of interest. Using these techniques, the authors identified that around 75% of the total vascular resistance in the heart resides in vessels beneath 200 μm (39). Subsequent work from this group has used the same technique to successfully measure cardiac microvascular dynamics in response to α_1_- and α_2_-adrenergic stimulation (42) and the role of nitric oxide in the response to adenosine (43).

## *In vitro* Imaging of the Heart—Thinking Outside the Box

While the above approaches were relatively successful in achieving their goal of tissue stabilization, it is fair to say that they were not easily accessible to all laboratories. The development of the tools described were often complex and not always applicable across all use cases. In these situations, many users turned to the Langendorff heart, or isolated perfused heart, as a potential means for cardiac tissue imaging but with the ability to more easily control some of the movement aspects of the tissue.

The Langendorff model was first conceptualized by Langendorff (44), and since then has become a mainstay of the cardiovascular research community. However, unlike many other techniques which have their origins based in long history, the basic methodology for the Langendorff model remains largely unchanged since its original inception. For a more detailed and comprehensive description of the technique, the authors are directed to an excellent review covering this topic (45). However, to summarize—the heart is removed via careful dissection and the aorta is cannulated for the (retrograde) administration of a perfusion buffer. Administering this buffer against the normal physiological direction of flow closes the aortic valve and forces fluid to flow via the left and right main coronary arteries and into the resulting microvasculature. While the Langendorff model has some obvious disadvantages, there are some key advantages to consider: the model is straightforward, low cost, reproducible, and allows for the study of the heart in isolation.

In almost all use cases of the Langendorff model, the heart is perfused with Krebs-Henseleit buffer (KHB) (45). This buffer primarily relies on glucose as the primary metabolic substrate for the working heart. While this is sufficient to cover the energy needs of the tissue, perfusion with KHB (or other physiological buffers) does significantly limit the use of the Langendorff preparation for *in vitro* modeling of leukocyte trafficking using microscopic techniques. It should be noted that the Langendorff heart can be perfused with blood and interestingly, a number of studies have shown that doing this does significantly improve the function of the heart in this model (46–48). In smaller animals, the volumes of blood required for constant perfusion of this model may be prohibitive; perfusing one murine heart would require blood from many donors (49). In addition, blood from larger species is often unsuitable as their relatively larger erythrocytes are less able to traverse murine capillaries (49). There are also potential questions about the validity of using these models for immune cell trafficking; studies have shown that blood collection in itself can result in leukocyte activation in the resulting isolate (50). In addition, oxygenation of isolated blood (a critical requirement) can often lead to cellular damage due to the formation of foam (51). Unfortunately, the relative expense and complication of using blood vs. KHB has meant that most researchers—unless they specifically require blood supplementation—favor KHB or other physiological buffers over perfusion with haematopoietic components. Some more complex options exist in order to facilitate blood perfusion (for example, parabiotic models); the reader is directed to other reviews where this is considered in more detail (49).

Very few studies have used the *ex vivo* perfused heart for imaging immune cell trafficking. It should be noted that it is not the case that there are a lack of *ex vivo* perfused heart models in which immune cells are administered; there are such models—but rather that these models do not undergo live imaging, instead relying on immunostaining or scanning electron microscopy methods on cut sections (52–54). Kuppat et al. used widefield fluorescence imaging, at the terminal end of their experiment and after the inducement of cardioplegia, to identify neutrophil trafficking in the microvasculature. Administration of labeled neutrophils and FITC-dextran (for vascular contrast) into the perfused *ex vivo* heart has been used to examine the role of angiotensin I converting enzyme (ACE) and nitric oxide in the attenuation of inflammation post ischemia-reperfusion injury (55). Neutrophil recruitment was markedly enhanced in guinea pig hearts following ischemia-reperfusion (IR) injury, a phenomena which could be reduced by administration of cilazaprilat (an ACE inhibitor) or enhanced by treatment with nitro-L-arginine (NOLAG) (55). The authors were also able to demonstrate some capillary plugging in this model—a phenomena where excessive leukocyte recruitment results in the blockage of capillaries and an ultimate failure of perfusion; often, when this occurs following IR injury, this is termed “no-reflow” (56).

Given the potential benefits of the Langendorff system, it remains surprising that to date this model has not been readily used to dynamically monitor the mechanisms of cellular trafficking in the perfused heart. What makes this perhaps more surprising is that the Langendorff model has been used extensively for *imaging* of the heart but outside of the context of immune cell trafficking. There are many examples of the model being used for other investigations, such as: imaging of sarcomere length during cardiac mechanics (57, 58); calcium handling and the identification of various types of calcium waves during health and disease (59–62); and the assessment of mitochondrial function using dyes which are sensitive to mitochondrial membrane potential (ΔΨm) (63). Much of this imaging is actually performed live and used to generate image data for quantitative analysis; as such, the tools are readily available in most labs to adapt this model to examine cellular trafficking. Indeed, a combinatorial approach (for instance, imaging calcium transients alongside cellular recruitment) may help to yield more detailed information about the molecular events that occur in the context of immune cell recruitment. Given many labs will have access to heart tissue that is not suitable for intravital imaging but may be accessible for later *ex vivo* imaging, it may be worth considering the Langendorff model as a potential tool for investigating mechanisms of cell recruitment.

Other imaging models exist beyond than the Langendorff system, albeit somewhat less complex. For some experimental purposes, groups have found that imaging cardiac explants without any flow or contractile activity may provide some useful information about inflammatory processes. As a small part of a larger intravital study, Li et al. used cardiac explants to examine the behavior of neutrophils following IR injury (64). Hearts were isolated from LysM-GFP mice, in which neutrophils could be readily identified by their GFP^hi^ phenotype (64). Using two-photon microscopy, few neutrophils were identified in mice without IR injury; however, following IR injury, significantly increased numbers of neutrophils could be observed in the microvasculature. More importantly, even in the absence of any flow, these neutrophils could be observed crawling and transmigrating through the vessel wall into the tissue parenchyma (64). As an important methodological note, the authors were able to image the explanted heart for at least 6 h post reperfusion, likely due to the low phototoxicity and bleaching that is inherent with two-photon microscopy. However, the lack of flow and contraction in this model limits its translation to the whole organ.

## Confocal and Multiphoton Intravital Imaging of the Beating Heart—Seeing Things Differently

**Figure 1:**
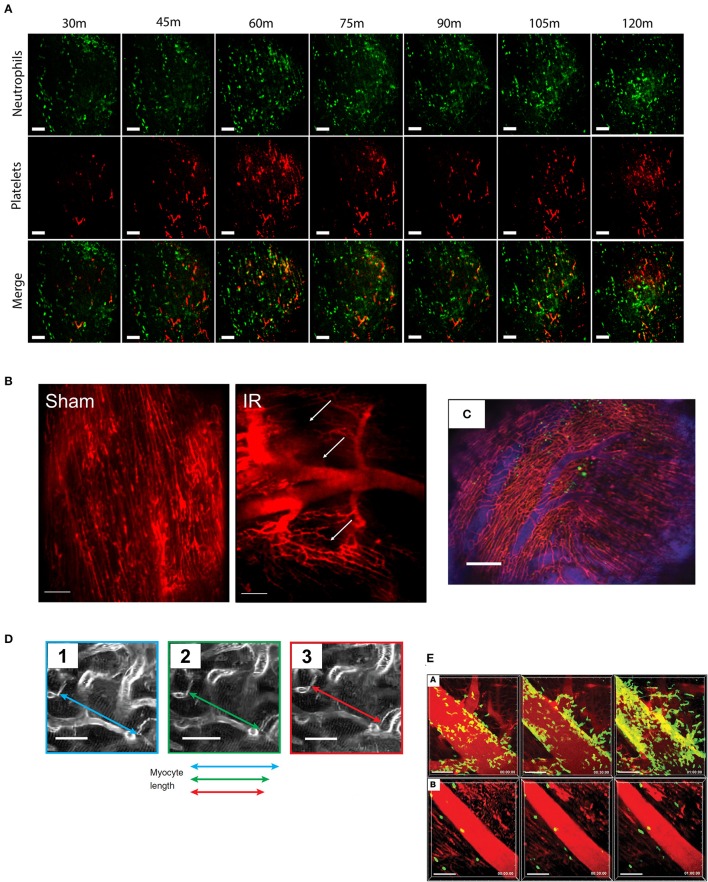
Typical imaging results from cardiac intravital microscopy. As the technique has developed, so has the variety of parameters that can be assessed from cardiac intravital imaging. **(A)** Using fluorescently labeled antibodies, the recruitment of leukocytes (green, anti-Gr-1) and platelets (red, anti-CD41) to injured heart can be monitored. Importantly, particularly in models of acute inflammation where understanding of the chronology of cell recruitment is crucial, imaging can be taken from the same area over a period of time. Cell recruitment can be counted by simple identification, while thrombus formation can be analyzed by masking upon positive signal. Figures adapted from published figure (70) (scale bar: 100 μm). **(B)** Perfusion of the mouse with a vascular contrast agent allows researchers to identify viable areas of tissue perfusion and permits the calculation of functional capillary density. During IR injury, for example, there is a clear reduction in the amount of patent microvessels compared to an animal undergoing sham surgery. Areas of “no-reflow” are shown with white arrows. Figures adapted from published figure (68) (scale bar: 100 μm). **(C)** Using a combination of vascular contrast agents and cellular staining, cell trafficking in and out of vessels can be examined in depth. The range of fluorescent channels which can be examined is limited only by the availability of filters and dyes. In this example, GFP-labeled bone marrow cells can be seen in the heart, both inside and outside of the vascular space. Adapted from Lee et al. (69) (scale bar: 200 μm). **(D)** Advancements in stabilization (and synchronization) techniques have allowed for detailed physical measurements to be performed due to the near eradication of motion artifacts. For example, imaging the contraction of single cardiomyocytes is now possible using this technique. Adapted from published figure (70) (scale bars: 20 μm). **(E)** The use of genetically modified animal strains facilitates studies that examine trafficking without the need for antibody (or tracker)-based staining techniques. In this example, LysM-GFP positive neutrophils can been seen trafficking to the heart following IR injury (subpanel **A**), which can be inhibited by administration of a CXCL2 neutralizing antibody (subpanel **B**). Adapted from published figure (71) (scale bars: 50 μm).

Cardiac intravital imaging advanced significantly with the introduction and uptake of more advanced imaging techniques such as laser scanning (raster scanning) confocal imaging, spinning disk confocal imaging, and multiphoton imaging. As understanding these techniques is key to garnering a full appreciation of how these new technologies have helped cardiac intravital reach the next level, we shall briefly touch on them from a technical stand point in this review an overview of the advantages and disadvantages of the methods discussed in this section is shown in Table 1. Should readers want further technical information about these techniques, there are excellent reviews to which one should refer (65–67). Examples of the types of images generated from cardiac intravital microscopy using these techniques are provided (Figure 1).

**Table 1:** Summary of the various options for cardiac intravital imaging.

	**Disadvantages**	**Advantages**
**Imaging method**		
Raster-scanning confocal	Inherently slower; requires advanced stabilization and/or gating mechanisms in order to use for cardiac IVM Prone to “tearing” artifacts due to the line-by-line nature of image formation	Identical illumination of each pixel (field uniformity) Well-established technique, more widely available, numerous vendors Better light transmission vs. spinning disk methods
Spinning disk confocal	Identical illumination of each pixel cannot be guaranteed (lack of field uniformity) Potentially increased background/non-focal plane light (due to pinhole crosstalk) Reduced light transmission vs. raster scanning methods	Generally much faster than raster scanning techniques Full field illumination; less prone to “tearing”-type artifacts (see Figure 2) Typically reduced laser powers are required; less photo-bleaching/-toxicity
**Stabilizer attachment mechanism**		
	**Disadvantages**	**Advantages**
Glue	Permanent; stabilizer cannot readily be manipulated once attached Requires care—excessive glue can block the field of view and removing it is difficult if placed incorrectly Potential for side effects from the application of glue	More straightforward than suction; not reliant on external equipment Quicker than suction stabilization techniques Firm bonding to the stabilizer
Suction	Requires monitoring to ensure seal is maintained Potential for tissue damage with high levels of suction Disturbance of tissue perfusion underneath the suction ring	Reversible and can be moved if positioned incorrectly Less impact on the tissue surface for post-imaging experiments
**Gating**		
	**Disadvantages**	**Advantages**
No gating	Highest potential for motion artifacts in resulting images Requires large numbers of frames to ensure sufficient data for analysis; inefficient data capture method Uses low exposure times and thus needs (relatively) stronger staining	Surgically more straightforward; ECG/respiratory readings not needed Technically more straightforward than gating mechanisms; less specialist knowledge required Quicker set up time
Retrospective	Requires large numbers of frames to ensure sufficient data for analysis; inefficient data capture method More technically challenging than not gating If pacing, potential for pacing to interfere with experimental outputs Requirement for post-capture processing	Better mechanism for abrogating motion artifacts than not gating Requires less complex/reactive equipment than prospective gating
Prospective	Most technically challenging gating option If pacing, potential for pacing to interfere with experimental outputs Requires non-trivial technical equipment to process a number of incoming input sources	Best mechanism for abrogating motion artifacts More efficient capture technique than retrospective gating or no gating Post-capture processing not required

*There are a variety of options available to researchers who wish to begin experiments using cardiac intravital imaging. The above table summarizes some of the advantages and disadvantages of the various amendable options in this experimental model*.

**Figure 2:**
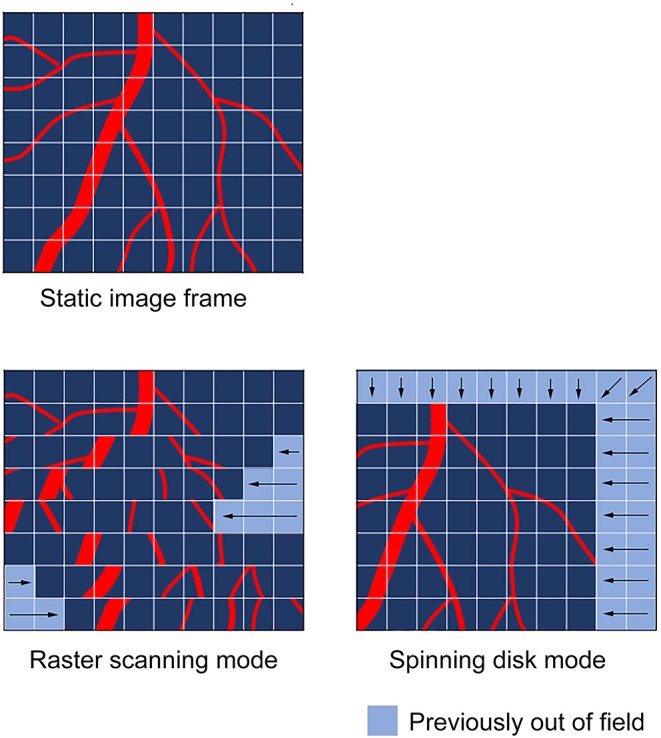
Common artifacts observed during cardiac intravital in raster and spinning disc scanning modes. The type of motion artifacts observed during cardiac intravital are highly dependent on the nature of the scanning mode utilized to obtain the images. Raster scanning modes, which use a scan head to move across the tissue in a point-by-point fashion are subject to “tearing” artifacts, which result from the tissue moving while the scan head progresses across the tissue. This manifests in scanlines being positionally out-of-sync as the scan head moves in its secondary axis. In spinning disk mode, the scan head images the whole field of view during the imaging procedure. Thus, spinning disk imaging modes tend to cause whole-field image shifts.

During regular widefield fluorescence imaging, the tissue is illuminated by a block of excitation light as it is focused in toward the plane of interest. As a result, not only does the excitation light excite the target focal plane, but also excites other focal planes above and beneath the target. These planes generate emission light, but as they are not in the same focal plane as the detector, they impact negatively on the resulting image and generally reduces image quality. Confocal imaging introduces a pinhole which rejects light that is not in the same focal plane as the detector. This significantly improves the image quality by rejecting out-of-focus light, reducing blur and improving resolution. There are two main methods relevant to this review: laser scanning and spinning disk. Although both are forms of confocal imaging, they should be considered very distinct in their methodologies. In the case of laser scanning confocal, this imaging is performed in a point-scanning mode; the microscope system has a single raster scanning beam which moves across the tissue to generate the resulting image pixel-by-pixel based on the returned signal at each point. Once reaching the end of the image, the scan head can either return to the start position and begin again (one-way) or reverse its direction (round trip). It may be immediately obvious that in a moving tissue, point-scanning methods may not be suitable for image capture. For the entire image frame to be captured in the exact same physical space, the tissue would need to remain entirely still for the duration of each raster scan duration. This—at least in the case of the heart—is impossible without complete cardioplegia; even with the most aggressive stabilization techniques, the heart retains some degree of contraction within the confines of the imaging window. Spinning disk confocal avoids some of these problems by generating multiple beams which illuminate the tissue simultaneously. These multiple beams are generated by placing a spinning disk (which contains up to 20,000 pinholes) in the emission light path. This disk runs at up to 10,000 rpm and illuminates the entire sample with these generated beams. The use of a second disk, running in sync with the first provides the pinhole effect that is critical to confocal imaging and allows for the generation of confocal quality imaging, but without any loss of speed or image quality due to raster scanning methods. As a result, the raw (unprocessed) images derived from these techniques differ greatly (Figure 2). Raster scanning techniques tend to generate intravital imaging that has “tearing” artifacts, where imaging fields have intra-field artifacts due to movements during the travel of the scan head. On the other hand, spinning disk techniques tend to have inter-field artifacts, stemming from movements of the entire field between frames. In addition, in contrast to raster scanning techniques, field uniformity (identical stimulation at each pixel of the image) cannot be guaranteed. While this may be an issue for models that rely on pixel intensity (such as platelet recruitment), it can be mitigated by averaging frames across an imaging window (rather than analyzing still images). Spinning disk confocal is also prone to a phenomenon known as pinhole crosstalk. This results from out-of-plane/scattered emissions passing through an adjacent pinhole on the disk which is not in alignment with the pinhole the excitation light passed through (72). This effect leads to an increased background signal on spinning disk confocal microscopy when compared to laser scanning confocal. However, the speed advantages gained from employing spinning disk confocal has allowed some labs—including ours—to perform cardiac intravital without necessarily needing aggressive post-analysis image restoration techniques (68).

Multiphoton imaging is a further and more powerful form of confocal imaging that delivers focally limited imaging but without the need for a pinhole. In contrast with other microscopy techniques which use continuous wave laser/light sources, multiphoton microscopy uses pulsed lasers to generate a stream of infrared photons. As infrared photons have less energy than their non-infrared counterparts, the near simultaneous (within 1 × 10^−18^ s) absorption of two photons at the same fluorophore is required for excitation (as the wavelengths used are infrared, excitation wavelengths used for fluorophores are longer than they would be using confocal microscopy). In addition, as the beam is focused through the microscope optics, the photons become more and more crowded in space—thus increasing the chance that two photons may simultaneously interact with a single fluorophore. The net result of this combination of factors is that there is a small volume downstream of the objective—the focal plane, in this case—where the probability of two photons meeting a fluorophore near-simultaneously is sufficiently high enough that excitation can occur. It is this physical phenomenon that results in the excitation of fluorophores only within the focal plane—there is no out of focus excitation and it is this which delivers confocality without the need for a pinhole. Similar to laser scanning confocal, multiphoton microscopy operates in a point-scanning mode, working across the tissue in a pixel-by-pixel fashion. Again, similar to laser-scanning confocal, multiphoton imaging is highly sensitive to movement and requires a mixture of physical stabilization, a degree of predictability in tissue movement (possibly including gating—discussed later), and post-acquisition image manipulation.

The first studies to effectively develop cardiac intravital using these imaging techniques to monitor immune cell trafficking in real-time were published at around the same time in 2012. The first of these studies, by Li et al. (64), used multiphoton microscopy to identify some of the basic characteristics of leukocyte trafficking within the heart, in real-time. This paper used two main approaches, combining elegant surgery, and an external stabilization device. For the first approach, the authors connected a donor heart into the circulation of a recipient mouse. To achieve heterotopic heart transplantation, they engrafted donor hearts into the right cervical region of recipient animals. In order to generate injury, some donor hearts had undergone cold (4°C) ischemia for 1 h prior to reconnection. To re-establish perfusion, the donor ascending aorta and pulmonary artery were connected to the recipient's right common carotid artery and right external jugular vein, respectively. Once successfully transplanted, the donor heart adopted a normal heartbeat in its ectopic position. In order to image this heart in position, the authors developed an imaging chamber consisting of a glass coverslip which was brought into contact with the heart using height-adjustment dials. The authors also applied a small ring of veterinary adhesive (cyanoacrylate based) in order to physically stabilize the heart against the chamber glass. By engrafting wild-type (WT) donor hearts onto either LysM-GFP mutant mice (whose neutrophils express high levels of GFP) or CX3CR1 GFP/GFP mutant mice (B6.129P-Cx3cr1^tm1Litt^/J; whose monocytes express GFP), the authors were able to image the trafficking of exogenous (non-resident) neutrophils and monocytes to the heart during reperfusion (Q-dots were used as a vascular contrast medium). Using this technique, the authors identified minimal neutrophil recruitment in post-capillary venules during IR injury, but rather localized most recruitment activity to the walls of the larger coronary veins where they identified intraluminal crawling and congregation. In addition, monocyte recruitment to the microvasculature was also identified, although congregation of monocytes was not identified in this model (65). Perhaps one of the most interesting capabilities of this model is the ability to graft donor hearts from mutant mice; allowing researchers to investigate cardiac-specific mechanisms in the context of otherwise normal systemic physiology. The authors capitalized on this, using donor hearts from ICAM-1 mutants (B6.129S4-Icam1^tm1Jcgr^/J); these mice do not express a functional form of ICAM-1 (73). Results from these hearts, alongside the use of Mac-1 blocking strategies, suggested a key role for Mac-1/ICAM-1 in intravascular crawling and transmigration of neutrophils. Subsequently, the authors adapted their original stabilization technique to stabilize the heart in its normal intrathoracic position. No neutrophil recruitment was noted in the absence of inflammation. Importantly, results from heart imaging in its normal position *in situ* correlated with the results obtained from heterotopic heart transplants. The authors noted that in this model, mice were able to tolerate this imaging procedure for at least three hours; this is important to note, as this preparation did not use pacing, or any special ventilation techniques (such as supplementation with medical air). The authors have subsequently used this imaging model in further sophisticated studies, including: the identification of a critical role for CCR2^+^ monocytes in driving neutrophil recruitment following ischemia (71) (imaging from this study is included in Figure 1E); showing evidence that tissue-resident macrophages promote or inhibit monocyte recruitment dependent on their CCR2 expression phenotype (74); and that ferroptosis (an iron-dependent form of cell death) is a key mediator of neutrophil recruitment in transplanted hearts through a TLR4/TRIF/Type I IFN signaling pathway (75).

Later in 2012, Lee et al. published (69) an alternative method for intravital imaging of the cardiac microcirculation, utilizing not only tissue stabilization but also cardiorespiratory gating. On the former point, the stabilization proposed by Lee and colleagues was significantly less intensive than in the work of Li et al. (64). The stabilizer in this study was essentially a small ring machined from stainless steel, with an outer and inner diameter of 3.6 and 2.2 mm, respectively. This ring had a small groove on the base, and was designed to be attached to the heart surface, using veterinary adhesive. The stabilizer was attached to an arm which itself was attached to a manipulator allowing it to be advanced or adjusted to the correct location on the surface of the heart. Due to the relatively small internal diameter of the ring, the initial design was specifically for use with “stick” objectives—extremely thin objective lenses that were designed to fit within the center of the machined ring (these objectives, manufactured by Olympus, are now hard to reasonably hard to obtain). The authors also published a modification that could be applied to the stabilizer that allowed for the use of standard diameter water-immersion lenses (in this study, the x20 XLUMPLANFL, which has a lens diameter of 5.2 mm at its base and 10.5 mm where it meets the base of the objective). These standard objectives still permitted the capture of high-quality multichannel imaging (an example from this method can be seen in Figure 1C). One of the useful aspects about using such a small stabilizer and the ability to manipulate the stabiliser's location, is that the heart can be positioned such that the only pressure the heart experiences is that of it contracting against the stabilizer. The authors suggest that this is “similar as the heart beating against the chest wall” (69). Although stabilization provided mechanical support to the beating heart, motion artifacts remained and physiological gating became necessary in order to circumvent these issues. This gating was designed to retain capture fields only during specific points of the cardiac and respiratory cycle. Initially, the authors had considered (and attempted) gating imaging on points in the cardiac cycle alone. However, the authors noted that gating on the cardiac cycle alone was insufficient as pulmonary movements were also significant contributors to the physical movement of the heart. In order to eliminate movement, the authors chose two points on both cycles in which to capture images. In the cardiac cycle, they chose a 15 ms window after the appearance of the *P* wave, as measured by electrocardiogram (ECG). In the pulmonary cycle, a window of 90 ms near the end of expiration was selected; as the animals were mechanically ventilated, the observers could be sure of the exact point in respiration at which imaging would be triggered. The net result was the generation of an optimal imaging window during which image data could be retained. Using this information, the group designed an algorithm which could, in real-time, rebuild images using only line scans obtained during the optimal imaging window. Rebuilding these, the authors were able to generate captures in real-time, devoid of motion artifacts. In the initial paper, this model was tested, perhaps unsurprisingly, by examination of leukocyte recruitment following cardiac ischaemia-reperfusion injury. The authors labeled endogenous leukocytes by administration of Rhodamine 6G and noted leukocyte rolling in cardiac capillaries. The descriptions of leukocyte trafficking in this study were relatively preliminary and minimal analysis was performed on the trafficking described. Subsequent work from the group explored the addition of cardiac pacing as a means to gate imaging more reliably (76). The authors also suggested that this should also to allow for imaging to be better gated prospectively. A number of years later, the same group published an extensive methods paper, describing the various techniques they had developed to date in detail and distributing the 3D STL files in order for research groups to be able to print the stabilizers for themselves using 3D printing techniques (70). It is worth noting that others have since designed glue-dependent stabilizers that are relatively similar in design (77); but it is probably the case that are only so many ways that one can design a stabilizer for cardiac imaging—as the technique becomes more widespread, we would posit that significant differences in the overall design of stabilizers are unlikely.

We have also recently published work using a glue-based stabilizer for cardiac intravital microscopy (68). Although we have used a stabilizer that is similar in design to those used elsewhere in the literature, we have not used cardiopulmonary data to gate our imaging. Rather, we have used a combination of spinning disk confocal imaging, minimal exposure times, and high sensitivity detection in order to allow us to capture as many frames as possible during any given amount of time. The net result is that we have enough frames from which we can disregard those which are afflicted by motion artifacts, and retain those that are sufficiently clear (we have written software to help us achieve this, which we will come onto later). Using this technique, we have been able to examine the role that stem cells play in the downregulation of inflammatory injury following ischaemia-reperfusion injury. We have shown previously that haematopoietic stem cells (HSCs), in addition to their normal physiological role, are able to play an active role in the reduction of an acute inflammatory injury (78). Indeed, HSCs express a number of receptors for pro-inflammatory cytokines, such as the TNF receptors TNFR1 and TNFR2 (78), so it is contemplable that they may possess the ability to respond to inflammatory cytokines. Using spinning disk confocal imaging, we sought to identify the acute time course of neutrophil and platelet recruitment following the onset of reperfusion injury; and secondly, whether the administration of HSCs is able to affect the onset of this inflammatory response. By utilizing endogenous antibody labeling, we identified that both neutrophil and platelet recruitment is enhanced rapidly during reperfusion (a representative example of this imaging can be seen in Figure 1A). Furthermore, the administration of HSCs significantly reduced neutrophil and platelet accumulation during reperfusion. At the end of each experiment, we administered a vascular contrast agent to identify viable perfused microcirculation; as we could do this in the same animals, this allowed us to obtain additional functional data while not increasing the number of animals required for each group. Using this technique, we identified that IR injury is characterized with clear areas of microvascular no-reflow, evidenced by portions of cardiac microcirculation which do not take vascular contrast agent upon administration (a representative example of this imaging can be seen in Figure 1B).

Not all published methods rely on glue to affix stabilizers to the heart. Rather than rely on VetBond and other tissue adhesives, some groups have turned to negative pressures (suction) in order to affix their stabilizer devices to the tissue surface. Before suction stabilization was adopted as a means for imaging the heart, it had been used extensively as a means for imaging the lungs (79–81). Vinegoni et al. were the first to describe a suction based device for intravital imaging of the cardiac microvasculature (82). The device is small, similar to that described in Lee et al. (69); the internal chamber of the stabilization device has a diameter of 2 mm, while the outer diameter is 4.5 mm. The outer chamber is a hollow ring, which is attached to a vacuum regulator. This regulator provides gentle suction to the device and maintains the connection between the stabilizer and the tissue surface. The small diameter of the internal chamber does mean, again, that the user is highly dependent on access to micro-lens (or “stick”) objectives. However, suction based techniques have a very clear advantage—it is much, much easier to remove a stabilizer that is being held on by suction than it is to remove one that is affixed to the heart with glue. As such, using suction-based stabilization techniques allow for the movement of the stabilizer unit to different parts of the heart and/or the possibility of recovery surgery in cardiac intravital imaging models. Again, in this study, the authors utilized cardiopulmonary gating in order to attempt to remove motion artifacts. Critically, the authors go to some lengths in this study to convince that the application of the suction stabilizer leaves no lasting effect on the cardiac tissue. As a result of application, the authors show no visible cardiac damage, no change to ECG traces, and no macro- or microvascular damage identified by Griffonia simplicifolia-I lectin administration (which labels endothelial cells) (82).

An elegant approach was devised by Jung et al. who created a small suction-assisted endoscope which could be advanced into the chest cavity and toward the heart with minimally invasive surgery (83). The aim of the endoscope was to transmit signal from the base of an imaging probe to the top, such that an objective lens could image the top of the probe as if it were imaging the surface of the heart. By attaching a number of rod lenses together, the authors were able to achieve this, generating a lens with a x1 magnification and a length of 20 mm. The bottom two-thirds of this lens was housed within a steel sleeve, and surrounded by an outer steel tube which acted as the suction tube. This suction tube was connected at its end to a rubber tube which provided the suction source (at a pressure of 50 mmHg), with the upper third of the imaging probe protruding through the suction tube. This entire construct is then held in place, and imaged through the tip of the imaging probe with a high-numerical aperture objective. Validation data with the technique showed that application of suction through the construct virtually eliminated all movement, while the flow rate of cells through the microcirculation was not perturbed even at negative pressures as high as 150 mmHg (83). Furthermore, tissue damage was only observed at extreme levels of suction (300 mmHg) and no local inflammation was noted in areas where the suction was applied. One interesting aspect of this technique is that the authors were able to reproducibly (and with no evidence of damage) move the endoscope around the heart in order to image an area wider than a single field of view. This “wide-area scan” mode allows for individual captures to be tiled in a mosaic fashion, generating fields much larger than the initial limited field of view. In the publication associated with this work, the authors—using individual 250 μm fields of view—were able to construct an interlaced high resolution image of 1.2 mm by 1.2 mm (83). The authors went on to examine the recruitment of CX_3_CR1^+^ monocytes and neutrophils to the post ischaemic heart using both longitudinal (0, 1, or 6/7 days) and acute imaging (0, 5, or 30 min). Neutrophil recruitment increased acutely, with significantly enhanced neutrophil recruitment observed within 30 min [we have seen an identical observation using spinning disk confocal (68)]. Interestingly, neutrophil recruitment returns to baseline at 7 days post reperfusion. On the other hand, monocyte recruitment increases much more rapidly in reperfusion and precedes neutrophil recruitment, with a significant increase in monocyte presence as early as 5 min post reperfusion. Furthermore, this increase continues at 30 min and persists up to 6 days post-reperfusion. The authors also noted an increase in the number of monocytes flowing through the heart during reperfusion, but with a significant decrease in rolling during this period.

More recently, others have developed alternative suction stabilization techniques that do not depend on “stick” objectives or rod lenses. Matsuura et al. (84) have developed a suction-based stabilizer for use on rats that has a small central hole. In a slightly higher plane than the base of this central hole is an outlet for suction. Up from this is a notch onto which a larger cover slip sits, allowing the formation of a fully closed pressurized system once negative pressure is applied via suction through the outlet. The rat is positioned underneath the central hole of this stabilizer, and either side of the animal, two arms which protrude from the central portion of the stabilizer lock into two locking nuts which are located on metal breadboard and tightened to (a) lower the stabilizer in the z-plane onto the tissue, and (b) lock the stabilizer in place. With the coverslip in place between the tissue and the objective, water immersion objectives can be used with relative ease. Using a mixture of alignment and image processing techniques, the authors were able to monitor the trafficking of leukocytes in subsequent image frames, with frame to frame captures allowing for the calculation of leukocyte velocity and displacement. The authors were also able to identify an increase in leukocyte recruitment as a result of ischemia-reperfusion injury, which was induced via the insertion and inflation of a PCI balloon into a loop placed around the left anterior descending coronary artery. The authors further stated that leukocyte blockage of capillaries occurred within 1 h of reperfusion (84), consistent with results we have seen in our work (68).

## Nothing Alive Is Truly Still—Acquisition Data Processing and Adaptive Focusing Mechanisms for Imaging the Heart

While stabilization techniques are able to eradicate the vast majority of tissue movement, it is impossible (unless the tissue is rendered cardioplegic) to prevent all movement of stabilized cardiac tissue. This is because even within the central imaging window of a cardiac stabilizer, the myocardium will continue to contract, regardless of much gross movement is reduced. Therefore, further compensatory techniques are required to eliminate these movement artifacts from resulting data. At present there are two main mechanisms by which this can be achieved; either by the use of software processing, or adaptively focused optics. On the latter, a number of techniques now exist which are set up to allow microscope optics to move in line with tissue movement. One of the earliest iterations of this approach used a high-speed (~955fps) camera to detect movement of bright fluorescent particles in an imaging field, and subsequently moved the microscope objective accordingly to maintain these particles in the same initial location (85). This study was primarily for the movement of the objective in the *x,y* planes and not *z*. Additional work by the same authors later added the ability to also compensate for movement in the *z* plane, creating a system that could compensate for tissue movement in all three dimensions (86). Other solutions exist to help imaging setups mitigate against the movement of tissues in the axial plane. Spectral domain optical coherence tomography (OCT) has been used to rapidly measure the distance between a moving tissue and an imaging objective (87). When coupled with fast processing and piezoelectric control of objectives, this technique has been shown to be able to rapidly compensate for fast moving tissues in live animals.

Software processing is a much more accessible means for minimizing movement artifacts, from both a financial and a technical perspective. Software processing can be done either prospectively or retrospectively. Prospective image processing involves using information from the preparation and/or model to manipulate image capture in order to capture image frames that do not suffer from movement artifacts. In retrospective image processing, the image data is handled after it has been captured. In some cases, the data may be captured with a range of ancillary data such as ECG and respiratory cycles, while in others, image data may be captured alone and processed without the support of additional data sets. Image processing based on physiological parameters normally relies on either cardiac ECG gating, pulmonary gating, or a mixture of both. Such techniques aren't particularly new. In as far back as 1981, researchers were using the peak of the QRS complex as a timing reference for the triggering of an external stroboscopic light source (37). Although this is not technically a triggering of image capture (in this instance, the presence of light on a given image frame allows the non-illuminated frames to be ignored), modern day equivalents are essentially more efficient versions of the same technique. Much of the work in this area has developed in the lab of Ralph Weissleder et a. who have published a number of studies [including an excellent and comprehensive methods paper (70)] which detail extensively how they have applied these techniques. For prospective image gating, the ECG and/or respiratory data must be processed and linked directly to the capture hardware in real-time. Windows are set in which tissue movement is likely to be minimal—in most cases this is normally defined as the entire QRS complex for the ECG, and either the P_plat_ or PEEP segment for pulmonary airway pressure (70, 88, 89). Although prospective gating strategies are more efficient in that they require the collection of less data (i.e., data likely to be spurious is rejected even before it is captured), they also require more expensive hardware and for capture systems to be compatible with fast triggering. However, one system—prospective sequential segmented microscopy (PSSM)—has been used as a method for generating motion-artifact free intravital imaging of the heart (76). In this technique, cardiac pacing is used to provide absolute definition of the cardiac cycle. Using this known pacing information, image capture can be accurately harmonized with an appropriate point in the cardiac cycle (in this study, pulmonary movement was minimized by either electronic triggering of the ventilator or pausing of its activity). In PSSM, the capture frequency and the pacemaker frequency are slightly offset. By doing this, it is possible to capture images from the heart during the entire cardiac cycle. This technique has been used to make determinations of cardiomyocyte activity at the single cell level; data from this technique shows it is possible to image the contractile activity of individual cardiomyocytes (76) (an example of the imagery underlying this technique is shown in Figure 1D).

It is much more common to see retrospective image processing techniques used in the context of cardiac intravital microscopy. While some of these techniques have been designed specifically with cardiac intravital in mind, others have been designed more generally for intravital microscopy. Others are simply general image processing techniques which can be applied to intravital microscopy. We will cover briefly the latter as they are the most generic and not cardiac specific. General image processing in the context of cardiac intravital most often takes the form of registration and alignment tools. Image registration is a technique to align or reposition images that have shifted in respect to one another. It is most often used in medical scanning, such as in CT and MRI scanning, but does have a use in intravital when handling images with reasonably manageable movement artifacts. Numerous plugins are available either for use in ImageJ/Fiji [we have found SPIM Registration to be a good tool in our hands (90)] or standalone [*elastix* is a well-established tool in this regard (91, 92)]. Some specific tools have been designed for registration of intravital microscopy data. *IMART* (Intravital Motion Artifact Reduction Tool) is a software tool designed specifically for the removal of motion artifacts (via registration) of intravital microscopy imaging (93). This image registration tool takes advantage of the fact that sequential frames in a time series are likely to be very similar to one another, while still being able to compensate for noise due to things like the introduction of cells, vascular dyes, and antibodies (93). *StabiTissue* is another tool that can perform similar functions and was also specifically designed for the purposes of intravital microscopy (94). Registration tools are useful, in particular when stabilization has been used and small amounts of residual movement remain. This is often the case, we have found, when using spinning-disk confocal imaging techniques for cardiac intravital imaging. Of course, registration tools are not particularly useful for line scanned images unless the image data has been processed by another tool first to repair distortions due to “during-scan” movement.

Retrospective image processing tools have two different tasks depending on the type of image data they are processing; for instance, line/raster scanned image data is very different to spinning disk confocal image data. The latter, providing it has been captured at a fast-enough speed, will contain full fields of valid image data. The former will contain image frames which will be made up of individual line scans that will be out of sync in the *x,y* plane. In order to process this data, the software must be able to return to the ECG/lung airway pressure data, connect this data with line scans, and reject line scan data which falls outside of the cardiac/pulmonary windows. Indeed, this is the approach that most studies have taken in this regard (69, 70, 88). While this means that the constructed image will be made of individual line scans with different temporal profiles, the image capture rate should be sufficient that the differences between them are relatively (and more importantly, biologically) insignificant. It is important, if it possible, for researchers to capture both ECG data and lung airway pressure—movement of the lungs is a clear contributor to the movement of the heart and a failure to take this into consideration can cause serious difficulties for cardiac intravital imaging—particularly for line-scanning techniques. However, not all techniques obtain respiratory or cardiac physiology data during capture. Indeed, we have found that cardiac intravital using spinning disk confocal does not require ECG or respiratory gating to generate useable imaging. In these cases, retrospective processing of image data sets without this information is required. In these cases, the most frequent approach used for this type of processing is frame rejection, where those with significant motion artifacts are removed from an image set. There are a number of tools available to allow researchers to achieve this, which work in a broadly similar fashion with some slight differences in how they achieve their goal of frame rejection.

**Figure 3:**
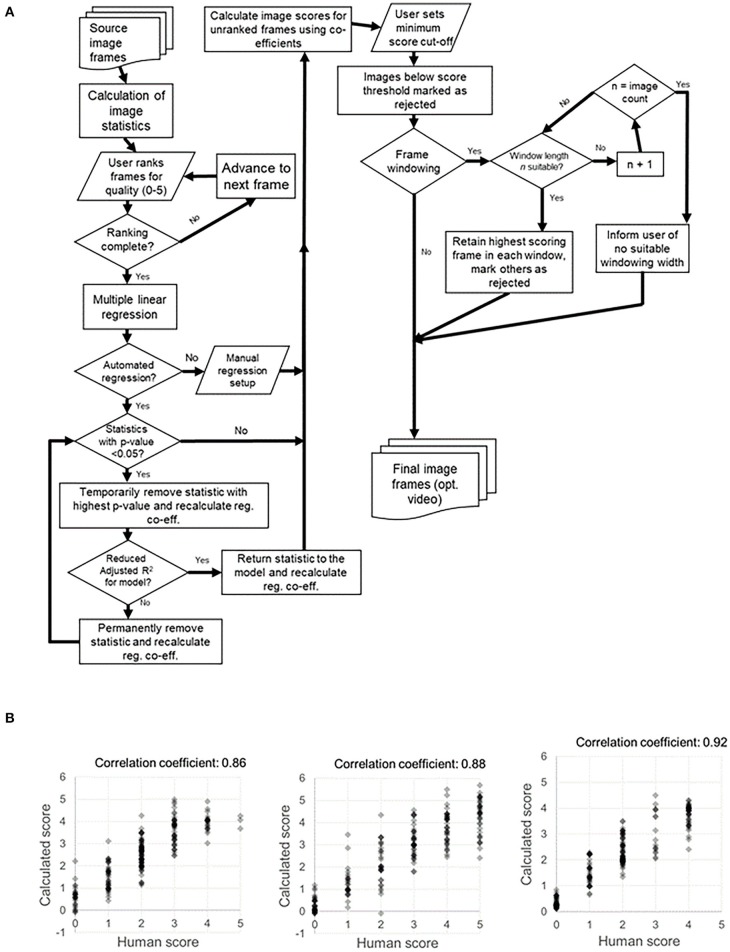
Process map for *Tify* and example of human vs. computed image scoring for example sets of images. **(A)** Our image processing tool Tify has a set procedural pathway for processing video captures from spinning disk confocal imaging. This quality-based tool is able to exclude frames from a large image stack by exclusion based on quality scoring. Using a small subset of human scores, Tify is able to use regression to calculate estimated scores for frames which it has not seen and subsequently exclude them from final output videos. **(B)** Crucially, Tify is able to calculate scores which are proximal to human scores.

Intravital_Microscopy_Toolbox is an ImageJ tool developed by Soulet et al. (95) which is designed to process an image stack and remove individual frames affected by motion artifacts. This software works by comparing each frame to a reference frame (or frames), and calculating a dissimilarity score. Using a cut-off value for this difference, the toolbox is able to remove frames which are considered to be too dissimilar to the reference frames. Along similar lines, we have developed a tool—termed *Tify*—which is able to process large image stacks and perform automated frame removal based on whether or not they meet given criteria (in terms of this review, this would be whether they contain motion artifacts or not) (96). However, rather than use reference frames, *Tify* instead uses a small subset of human scoring, underlying image statistics (such as standard deviation, entropy, and skewness), and regression analysis to attempt to “quality score” all frames from an entire image stack (for an idea of the methodology involved, see Figure 3). To make this software more applicable for intravital microscopy, we built in two additional statistics which were specifically relevant to cardiac intravital imaging: sum pixel ramp and segment intensity deviation. Detailed descriptions on how these statistics are calculated is beyond the scope of this review, but detailed information is available in the published manuscript detailing *Tify* (96). On average, users needed to score around 20 frames in order for *Tify* to score the remaining frames in the image stack with high levels of accuracy. To test this accuracy, we asked users to attribute a quality score (0–5) to 200 frames of a video obtained from cardiac intravital imaging. We then provided Tify with the scores from the first 20 of these, and assessed the software's accuracy at calculating the rest; the correlation co-efficient between human scores and calculated scores was high (e.g., 0.76 ± 0.03 for one set) (96). This approach has some advantages—because the image scores for unseen frames are calculated formulaically, *Tify* does not need to have seen images in advance in order to score them—it simply needs to have a formula with which to calculate the scores from. Therefore, it is possible—for instance—that a user could carry out intravital microscopy on the heart, perform scoring on those images, and generate a formula that can be used for all subsequent cardiac intravital microscopy images that are processed through *Tify*. One of the other advantages to *Tify* that, at least to our knowledge, is not available in other post-acquisition software, is the ability to retain temporal relationships between the retained frames. Most methods of frame removal do not rely on a simple system of cut-off; that is, if an image is below a certain quality of score, then it is removed from the stack. When we designed *Tify*, it was important to us that the output from our program retained some temporal relationship—for instance, if our software had disposed of 20 s of consecutive frames (an extreme example) then the result would have a large temporal gap in the output. To avoid this, we built in a feature called Frame Windowing, which rather than being a frame rejection technique, should rather be considered as a frame selection technique. Users specify the size of a window in *n* frames, and the software identifies the highest quality frame from within that window. Once done, the software moves onto the next *n* frames, and so on until the end of the input file. The resulting output is 1/*n* times the size of the original file, but each frame has some degree of temporal link between them.

## Concluding Remarks

For some time in the latter part of the 1970s and 1980s, it appeared as if cardiac intravital becoming a routine technique was an inevitability, given the pace of developments. It would be fair to comment that some of the techniques used in the 1980s were, even by todays technological standards, exceptionally elegant. However, the final leap toward using these techniques to monitor immune cell trafficking seemed to be lacking. It was not until the publications earlier this decade that allowed intravital microscopy of the heart to begin to gather pace again. As we approach the end of this decade, there are now a range of tools and approaches available to those who wish to explore intravital imaging of the cardiac microvasculature. We would put on record that such work is still challenging; the surgery required for exposure of the heart and induction of ischaemia is not trivial. However, the technological barriers that one used to face are no longer in the way. Using these new found techniques over the next decade, perhaps we may finally see an end to the days where the cardiac microcirculation is considered a research “black box.”

## Author Contributions

DK and NK wrote and drafted the manuscript.

### Conflict of Interest

The authors declare that the research was conducted in the absence of any commercial or financial relationships that could be construed as a potential conflict of interest.

## References

[1] British Heart Foundation Facts and Figures. (2019). Available online at: https://www.bhf.org.uk/what-we-do/our-research/heart-statistics/heart-statistics-publications/cardiovascular-disease-statistics-2019 (accessed 30 July, 2019).

[2] Bhatnagar P Wickramasinghe K Wilkins E Townsend N. Trends in the epidemiology of cardiovascular disease in the UK. Heart. (2016) 102:1945–52. 10.1136/heartjnl-2016-309573 27550425 PMC5256396

[3] British Heart Foundation 2019 Statistics Compendium. (2019). Available online at: https://www.bhf.org.uk/-/media/files/research/heart-statistics/bhf-statistics-compendium-2019-final.pdf?la=en (accessed 30 July 2019).

[4] Van Linthout S Tschöpe C Inflammation - cause or consequence of heart failure or both? Curr Heart Fail Rep. (2017) 14:251–65. 10.1007/s11897-017-0337-9 28667492 PMC5527060

[5] Seta Y Shan K Bozkurt B Oral H Mann DL. Basic mechanisms in heart failure: the cytokine hypothesis. J Card Fail. (1996) 2:243–9. 10.1016/S1071-9164(96)80047-9 8891862

[6] Torre-Amione G Kapadia S Benedict C Oral H Young JB Mann DL. Proinflammatory cytokine levels in patients with depressed left ventricular ejection fraction: a report from the Studies of Left Ventricular Dysfunction. (SOLVD). J Am Coll Cardiol. (1996) 27:1201–6. 10.1016/0735-1097(95)00589-7 8609343

[7] Kubota T Mctiernan CF Frye CS Slawson SE Lemster BH Koretsky AP. Dilated cardiomyopathy in transgenic mice with cardiac-specific overexpression of tumor necrosis factor-alpha. Circ Res. (1997) 81:627–35. 10.1161/01.RES.81.4.627 9314845

[8] Savvatis K Pappritz K Becher PM Lindner D Zietsch C Volk HD. Interleukin-23 deficiency leads to impaired wound healing and adverse prognosis after myocardial infarction. Circ Heart Fail. (2014) 7:161–71. 10.1161/CIRCHEARTFAILURE.113.000604 24300243

[9] Westermann D Van Linthout S Dhayat S Dhayat N Schmidt A Noutsias M. Tumor necrosis factor-alpha antagonism protects from myocardial inflammation and fibrosis in experimental diabetic cardiomyopathy. Basic Res Cardiol. (2007) 102:500–7. 10.1007/s00395-007-0673-0 17909696

[10] Xie F Fei X Zhang MB Zhang Y Wang HW Tang J. Quantitative evaluation of hepatic microvascular perfusion after ischemia-reperfusion injury in rabbits by contrast-enhanced ultrasound perfusion imaging. Ultrasound Med Biol. (2018) 44:1053–62. 10.1016/j.ultrasmedbio.2018.01.004 29478786

[11] Zeisberg EM Tarnavski O Zeisberg M Dorfman AL Mcmullen JR Gustafsson E. Endothelial-to-mesenchymal transition contributes to cardiac fibrosis. Nat Med. (2007) 13:952–61. 10.1038/nm1613 17660828

[12] Zhang Y Bauersachs J Langer HF. Immune mechanisms in heart failure. Eur J Heart Fail. (2017) 19:1379–89. 10.1002/ejhf.942 28891154

[13] Frantz S Hofmann U Fraccarollo D Schafer A Kranepuhl S Hagedorn I. Monocytes/macrophages prevent healing defects and left ventricular thrombus formation after myocardial infarction. FASEB J. (2013) 27:871–81. 10.1096/fj.12-214049 23159933

[14] Paulus WJ Tschöpe C. A novel paradigm for heart failure with preserved ejection fraction: comorbidities drive myocardial dysfunction and remodeling through coronary microvascular endothelial inflammation. J Am College Cardiol. (2013) 62:263–71. 10.1016/j.jacc.2013.02.092 23684677

[15] Likoff W Segal BL Kasparian H. Paradox of normal selective coronary arteriograms in patients considered to have unmistakable coronary heart disease. N Engl J Med. (1967) 276:1063–6. 10.1056/NEJM196705112761904 6025663

[16] Chilian WM. Microvascular pressures and resistances in the left ventricular subepicardium and subendocardium. Circ Res. (1991) 69:561–70. 10.1161/01.RES.69.3.561 1873859

[17] Matsumoto T Kajiya F Coronary microcirculation: physiology and mechanics. Fluid Dyn Res. (2005) 37:60–81. 10.1016/j.fluiddyn.2004.02.005

[18] Kassab GS Rider CA Tang NJ Fung YC. Morphometry of pig coronary arterial trees. Am J Physiol. (1993) 265:H350–365. 10.1152/ajpheart.1993.265.1.H350 8342652

[19] Steinhausen M Tillmanns H Thederan H. Microcirculation of the epimyocardial layer of the heart. I A method for *in vivo* observation of the microcirculation of superficial ventricular myocardium of the heart and capillary flow pattern under normal and hypoxic conditions. Pflugers Arch. (1978) 378:9–14. 10.1007/BF00581952 569829

[20] Yada T Hiramatsu O Kimura A Goto M Ogasawara Y Tsujioka K. In vivo observation of subendocardial microvessels of the beating porcine heart using a needle-probe videomicroscope with a CCD camera. Circ Res. (1993) 72:939–46. 10.1161/01.RES.72.5.939 8477527

[21] Toyota E Fujimoto K Ogasawara Y Kajita T Shigeto F Matsumoto T. Dynamic changes in three-dimensional architecture and vascular volume of transmural coronary microvasculature between diastolic- and systolic-arrested rat hearts. Circulation. (2002) 105:621–6. 10.1161/hc0502.102964 11827929

[22] Toyota E Ogasawara Y Hiramatsu O Tachibana H Kajiya F Yamamori S. Dynamics of flow velocities in endocardial and epicardial coronary arterioles. Am J Physiol Heart Circulat Physiol. (2005) 288:H1598–603. 10.1152/ajpheart.01103.2003 15550516

[23] Crea F Camici PG Bairey Merz CN. Coronary microvascular dysfunction: an update. Eur Heart J. (2014) 35:1101–11. 10.1093/eurheartj/eht513 24366916 PMC4006091

[24] Pries AR Habazettl H Ambrosio G Hansen PR Kaski JC Schachinger V. A review of methods for assessment of coronary microvascular disease in both clinical and experimental settings. Cardiovasc Res. (2008) 80:165–74. 10.1093/cvr/cvn136 18511433

[25] Pries AR Reglin B. Coronary microcirculatory pathophysiology: can we afford it to remain a black box? Eur Heart J. (2016) 38:478–88. 10.1093/eurheartj/ehv760 26843279 PMC5381591

[26] Lee S Courties G Nahrendorf M Weissleder R Vinegoni C. Motion characterization scheme to minimize motion artifacts in intravital microscopy. J Biomedi Opt. (2017) 22:36005–36005. 10.1117/1.JBO.22.3.036005 28253383 PMC5333764

[27] Wagner DD Frenette PS. The vessel wall and its interactions. Blood. (2008) 111:5271–81. 10.1182/blood-2008-01-078204 18502843 PMC2396724

[28] Marzi I Intravital microscopy of the liver for investigation of microcirculation, leukocyte-endothelial interactions and macrophage function - Experimental results after liver transplantation. Transplantationsmedizin. (1994) 6:91–8.

[29] Sumen C Mempel TR Mazo IB Von Andrian UH. Intravital microscopy: visualizing immunity in context. Immunity. (2004) 21:315–29. 10.1016/j.immuni.2004.08.006 15357943

[30] Pittet MJ Weissleder R. Intravital imaging. Cell. (2011) 147:983–91. 10.1016/j.cell.2011.11.004 22118457 PMC3824153

[31] Kavanagh DP Yemm AI Alexander JS Frampton J Kalia N. Enhancing the adhesion of hematopoietic precursor cell integrins with hydrogen peroxide increases recruitment within murine gut. Cell Transplant. (2013) 22:1485–99. 10.3727/096368912X653192 22889470

[32] White RL Nash G Kavanagh DP Savage CO Kalia N. Modulating the adhesion of haematopoietic stem cells with chemokines to enhance their recruitment to the ischaemically injured murine kidney. PLoS ONE. (2013) 8:e66489. 10.1371/journal.pone.0066489 23840488 PMC3686749

[33] Teo GS Yang Z Carman CV Karp JM Lin CP. Intravital imaging of mesenchymal stem cell trafficking and association with platelets and neutrophils. Stem Cells. (2015) 33:265–77. 10.1002/stem.1848 25263183 PMC4270897

[34] Martini J Honig CR. Direct measurement of intercapillary distance in beating rat heart in situ under various conditions of O 2 supply. Microvasc Res. (1969) 1:244–56. 10.1016/0026-2862(69)90026-0 5406306

[35] Tillmanns H Ikeda S Hansen H Sarma JS Fauvel JM Bing RJ. Microcirculation in the ventricle of the dog and turtle. Circ Res. (1974) 34:561–9. 10.1161/01.RES.34.4.561 4826931

[36] Nellis SH Leidtke AJ Pressures and dimensions in the terminal vascular bed of the myocardium determined by a new free-motion technique. In: Tillmanns H Kubler W Zebe H editors. Microcirculation of the Heart: Theoretical and Clinical Problems. Berlin: Springer-Verlag (1982). p. 61–73.

[37] Nellis SH Liedtke AJ Whitesell L. Small coronary vessel pressure and diameter in an intact beating rabbit heart using fixed-position and free-motion techniques. Circ Res. (1981) 49:342–53. 10.1161/01.RES.49.2.342 7249271

[38] Chilian WM Defily DV. Methodological approaches used for the study of the coronary microcirculation *in situ*. J. Vascul Res. (1991) 28:236–44. 10.1159/000158868 2001475

[39] Chilian WM Eastham CL Marcus ML. Microvascular distribution of coronary vascular resistance in beating left ventricle. Am J Physiol. (1986) 251:H779–788. 10.1152/ajpheart.1986.251.4.H779 3766755

[40] Yoder BA Siler-Khodr T Winter VT Coalson JJ. High-frequency oscillatory ventilation: effects on lung function, mechanics, and airway cytokines in the immature baboon model for neonatal chronic lung disease. Am J Respir Crit Care Med. (2000) 162:1867–76. 10.1164/ajrccm.162.5.9912145 11069828

[41] Evans E Biro P Bedforth N Jet ventilation. BJA Educ. (2007) 7:2–5. 10.1093/bjaceaccp/mkl061

[42] Jones CJ Defily DV Patterson JL Chilian WM. Endothelium-dependent relaxation competes with alpha 1- and alpha 2-adrenergic constriction in the canine epicardial coronary microcirculation. Circulation. (1993) 87:1264–74. 10.1161/01.CIR.87.4.1264 8384938

[43] Jones CJ Kuo L Davis MJ Defily DV Chilian WM. Role of nitric oxide in the coronary microvascular responses to adenosine and increased metabolic demand. Circulation. (1995) 91:1807–13. 10.1161/01.CIR.91.6.1807 7882491

[44] Langendorff O Untersuchungen am uberlebenden Saugethierherzen. Pflugers Arch Ges Physiol Mensch Tiere. (1895) 61:291–332. 10.1007/BF01812150

[45] Bell RM Mocanu MM Yellon DM. Retrograde heart perfusion: the Langendorff technique of isolated heart perfusion. J Mol Cell Cardiol. (2011) 50:940–50. 10.1016/j.yjmcc.2011.02.018 21385587

[46] Deng Q Scicli AG Lawton C Silverman NA. Coronary flow reserve after ischemia and reperfusion of the isolated heart. Divergent results with crystalloid versus blood perfusion. J Thorac Cardiovasc Surg. (1995) 109:466–72. 10.1016/S0022-5223(95)70277-6 7877307

[47] Podesser BK Hallstrom S Schima H Huber L Weisser J Kroner A. The erythrocyte-perfused “working heart” model: hemodynamic and metabolic performance in comparison to crystalloid perfused hearts. J Pharmacol Toxicol Methods. (1999) 41:9–15. 10.1016/S1056-8719(99)00018-0 10507753

[48] Liao R Podesser BK Lim CC. The continuing evolution of the Langendorff and ejecting murine heart: new advances in cardiac phenotyping. Am J Physiol Heart Circ Physiol. (2012) 303:H156–167. 10.1152/ajpheart.00333.2012 22636675 PMC3404701

[49] Sutherland FJ Hearse DJ. The isolated blood and perfusion fluid perfused heart. Pharmacol Res. (2000) 41:613–27. 10.1006/phrs.1999.0653 10816330

[50] Cotter MJ Norman KE Hellewell PG Ridger VC. A novel method for isolation of neutrophils from murine blood using negative immunomagnetic separation. Am J Pathol. (2001) 159:473–81. 10.1016/S0002-9440(10)61719-1 11485906 PMC1850545

[51] Faria M De Pinho MN Extracorporeal blood oxygenation devices, membranes for. In: Drioli E Giorno L editors. Encyclopedia of Membranes. Berlin; Heidelberg: Springer Berlin Heidelberg (2015). p. 1–19. 10.1007/978-3-642-40872-4_1468-1

[52] Okazaki Y Cao ZL Ohtsubo S Hamada M Naito K Rikitake K. Leukocyte-depleted reperfusion after long cardioplegic arrest attenuates ischemia-reperfusion injury of the coronary endothelium and myocardium in rabbit hearts. Eur J Cardiothorac Surg. (2000) 18:90–7. 10.1016/S1010-7940(00)00436-X 10869946

[53] Takarabe K Okazaki Y Higuchi S Murayama J Natsuaki M Itoh T. Nicorandil attenuates reperfusion injury after long cardioplegic arrest. Asian Cardiovascul Thor Annals. (2007) 15:204–9. 10.1177/021849230701500306 17540988

[54] Abicht JM Sfriso R Reichart B Langin M Gahle K Puga Yung GL. Multiple genetically modified GTKO/hCD46/HLA-E/hbeta2-mg porcine hearts are protected from complement activation and natural killer cell infiltration during *ex vivo* perfusion with human blood. Xenotransplantation. (2018) 25:e12390. 10.1111/xen.12390 29536572

[55] Kupatt C Habazettl H Zahler S Weber C Becker BF Messmer K. ACE-inhibition prevents postischemic coronary leukocyte adhesion and leukocyte-dependent reperfusion injury. Cardiovascul Res. (1997) 36:386–95. 10.1016/S0008-6363(97)00191-0 9534860

[56] Ragosta M Camarano G Kaul S Powers ER Sarembock IJ Gimple LW. Microvascular integrity indicates myocellular viability in patients with recent myocardial infarction. New insights using myocardial contrast echocardiography. Circulation. (1994) 89:2562–9. 10.1161/01.CIR.89.6.2562 8205665

[57] Bub G Camelliti P Bollensdorff C Stuckey DJ Picton G Burton RA. Measurement and analysis of sarcomere length in rat cardiomyocytes in situ and *in vitro*. Am J Physiol Heart Circ Physiol. (2010) 298:H1616–25. 10.1152/ajpheart.00481.2009 20228259 PMC2867435

[58] Botcherby EJ Corbett A Burton RA Smith CW Bollensdorff C Booth MJ. Fast measurement of sarcomere length and cell orientation in Langendorff-perfused hearts using remote focusing microscopy. Circ Res. (2013) 113:863–70. 10.1161/CIRCRESAHA.113.301704 23899961

[59] Minamikawa T Cody SH Williams DA. In situ visualization of spontaneous calcium waves within perfused whole rat heart by confocal imaging. Am J Physiol. (1997) 272:H236–243. 10.1152/ajpheart.1997.272.1.H236 9038943

[60] Hama T Takahashi A Ichihara A Takamatsu T. Real time *in situ* confocal imaging of calcium wave in the perfused whole heart of the rat. Cell Signal. (1998) 10:331–7. 10.1016/S0898-6568(97)00136-8 9692676

[61] Kaneko T Tanaka H Oyamada M Kawata S Takamatsu T Three distinct types of Ca < sup>2+ < /sup> waves in langendorff-perfused rat heart revealed by real-time confocal microscopy. Circul Res. (2000) 86:1093–9. 10.1161/01.RES.86.10.1093 10827140

[62] Rubart M Wang E Dunn KW Field LJ. Two-photon molecular excitation imaging of Ca2+ transients in Langendorff-perfused mouse hearts. Am J Physiol Cell Physiol. (2003) 284:C1654–68. 10.1152/ajpcell.00469.2002 12584115

[63] Matsumoto-Ida M Akao M Takeda T Kato M Kita T. Real-time 2-photon imaging of mitochondrial function in perfused rat hearts subjected to ischemia/reperfusion. Circulation. (2006) 114:1497–503. 10.1161/CIRCULATIONAHA.106.628834 17000908

[64] Li W Nava RG Bribriesco AC Zinselmeyer BH Spahn JH Gelman AE. Intravital 2-photon imaging of leukocyte trafficking in beating heart. J Clin Invest. (2012) 122:2499–508. 10.1172/JCI62970 22706307 PMC3386827

[65] Ustione A Piston DW. A simple introduction to multiphoton microscopy. J Microsc. (2011) 243:221–6. 10.1111/j.1365-2818.2011.03532.x 21777244

[66] Oreopoulos J Berman R Browne M. Spinning-disk confocal microscopy: present technology and future trends. Methods Cell Biol. (2014) 123:153–75. 10.1016/B978-0-12-420138-5.00009-4 24974027

[67] Bayguinov PO Oakley DM Shih CC Geanon DJ Joens MS Fitzpatrick JJ. Modern laser scanning confocal microscopy. Curr Protoc Cytom. (2018) 85:e39. 10.1002/cpcy.39 29927100

[68] Kavanagh DPJ Lokman A Neag G Colley A Kalia N. Imaging the injured beating heart intravitally and the vasculoprotection afforded by haematopoietic stem cells. Cardiovasc Res. (2019) 115:1918–32. 10.1093/cvr/cvz118 31062860 PMC6803816

[69] Lee S Vinegoni C Feruglio PF Fexon L Gorbatov R Pivoravov M. Real-time *in vivo* imaging of the beating mouse heart at microscopic resolution. Nat Commun. (2012) 3:1054. 10.1038/ncomms2060 22968700 PMC3622400

[70] Vinegoni C Aguirre AD Lee S Weissleder R. Imaging the beating heart in the mouse using intravital microscopy techniques. Nat Protoc. (2015) 10:1802–19. 10.1038/nprot.2015.119 26492138 PMC5380003

[71] Li W Hsiao HM Higashikubo R Saunders BT Bharat A Goldstein DR. Heart-resident CCR2(+) macrophages promote neutrophil extravasation through TLR9/MyD88/CXCL5 signaling. JCI Insight. (2016) 1:e87315. 10.1172/jci.insight.87315 27536731 PMC4985028

[72] Shimozawa T Yamagata K Kondo T Hayashi S Shitamukai A Konno D. Improving spinning disk confocal microscopy by preventing pinhole cross-talk for intravital imaging. Proc Natl Acad Sci USA. (2013) 110:3399–404. 10.1073/pnas.1216696110 23401517 PMC3587224

[73] Xu H Gonzalo JA St Pierre Y Williams IR Kupper TS Cotran RS. Leukocytosis and resistance to septic shock in intercellular adhesion molecule 1-deficient mice. J Exp Med. (1994) 180:95–109. 10.1084/jem.180.1.95 7911822 PMC2191562

[74] Bajpai G Bredemeyer A Li W Zaitsev K Koenig AL Lokshina I. Tissue resident CCR2- and CCR2+ cardiac macrophages differentially orchestrate monocyte recruitment and fate specification following myocardial injury. Circ Res. (2019) 124:263–78. 10.1161/CIRCRESAHA.118.314028 30582448 PMC6626616

[75] Li W Feng G Gauthier JM Lokshina I Higashikubo R Evans S Ferroptotic cell death and TLR4/Trif signaling initiate neutrophil recruitment after heart transplantation. J Clin Invest. (2019) 130:2293–304. 10.1172/JCI126428 PMC6546457 30830879

[76] Aguirre AD Vinegoni C Sebas M Weissleder R. Intravital imaging of cardiac function at the single-cell level. Proc Natl Acad Sci USA. (2014) 111:11257–62. 10.1073/pnas.1401316111 25053815 PMC4128110

[77] Jones JS Small DM Nishimura N. *In vivo* calcium imaging of cardiomyocytes in the beating mouse heart with multiphoton microscopy. Front Physiol. (2018) 9:969. 10.3389/fphys.2018.00969 30108510 PMC6079295

[78] Kavanagh DP Yemm AI Zhao Y Frampton J Kalia N. Mechanisms of adhesion and subsequent actions of a haematopoietic stem cell line, HPC-7, in the injured murine intestinal microcirculation *in vivo*. PLoS ONE. (2013) 8:e59150. 10.1371/journal.pone.0059150 23554986 PMC3595270

[79] Kuhnle GE Leipfinger FH Goetz AE. Measurement of microhemodynamics in the ventilated rabbit lung by intravital fluorescence microscopy. J Appl Physiol. (1993) 74:1462–71. 10.1152/jappl.1993.74.3.1462 8482691

[80] Looney MR Thornton EE Sen D Lamm WJ Glenny RW Krummel MF. Stabilized imaging of immune surveillance in the mouse lung. Nat Methods. (2011) 8:91–6. 10.1038/nmeth.1543 21151136 PMC3076005

[81] Presson RGJr Brown MB Fisher AJ Sandoval RM Dunn KW Lorenz KS. Two-photon imaging within the murine thorax without respiratory and cardiac motion artifact. Am J Pathol. (2011) 179:75–82. 10.1016/j.ajpath.2011.03.048 21703395 PMC3123791

[82] Vinegoni C Lee S Gorbatov R Weissleder R. Motion compensation using a suctioning stabilizer for intravital microscopy. Intravital. (2012) 1:115–21. 10.4161/intv.23017 24086796 PMC3786172

[83] Jung K Kim P Leuschner F Gorbatov R Kim JK Ueno T. Endoscopic time-lapse imaging of immune cells in infarcted mouse hearts. Circ Res. (2013) 112:891–9. 10.1161/CIRCRESAHA.111.300484 23392842 PMC3834270

[84] Matsuura R Miyagawa S Fukushima S Goto T Harada A Shimozaki Y. Intravital imaging with two-photon microscopy reveals cellular dynamics in the ischeamia-reperfused rat heart. Sci Rep. (2018) 8:15991. 10.1038/s41598-018-34295-w 30375442 PMC6207786

[85] Lee S Nakamura Y Yamane K Toujo T Takahashi S Tanikawa Y Image stabilization for *in vivo* microscopy by high-speed visual feedback control. IEEE Transac Robot. (2008) 24:45–54. 10.1109/TRO.2007.914847

[86] Lee S Ozaki T Nakamura Y *In vivo* microscope image stabilization through 3-D motion compensation using a contact-type sensor. In: 2008 IEEE/RSJ International Conference on Intelligent Robots and Systems. Nice (2008). p. 1192–7.

[87] Sherlock B Warren S Stone J Neil M Paterson C Knight J. Fibre-coupled multiphoton microscope with adaptive motion compensation. Biomed Opt Exp. (2015) 6:1876–84. 10.1364/BOE.6.001876 26137387 PMC4467716

[88] Vinegoni C Lee S Feruglio PF Marzola P Nahrendorf M Weissleder R. Sequential average segmented microscopy for high signal-to-noise ratio motion-artifact-free *in vivo* heart imaging. Biomed Opt Exp. (2013) 4:2095–106. 10.1364/BOE.4.002095 24156067 PMC3799669

[89] Vinegoni C Lee S Aguirre AD Weissleder R. New techniques for motion-artifact-free *in vivo* cardiac microscopy. Front Physiol. (2015) 6:147. 10.3389/fphys.2015.00147 26029116 PMC4428079

[90] Preibisch S Saalfeld S Schindelin J Tomancak P. Software for bead-based registration of selective plane illumination microscopy data. Nat Methods. (2010) 7:418–9. 10.1038/nmeth0610-418 20508634

[91] Klein S Staring M Murphy K Viergever MA Pluim JP. elastix: a toolbox for intensity-based medical image registration. IEEE Trans Med Imag. (2010) 29:196–205. 10.1109/TMI.2009.2035616 19923044

[92] Shamonin DP Bron EE Lelieveldt BP Smits M Klein S Staring M. Fast parallel image registration on CPU and GPU for diagnostic classification of Alzheimer's disease. Front Neuroinform. (2013) 7:50. 10.3389/fninf.2013.00050 24474917 PMC3893567

[93] Dunn KW Lorenz KS Salama P Delp EJ. IMART software for correction of motion artifacts in images collected in intravital microscopy. Intravital. (2014) 3:e28210–e28210. 10.4161/intv.28210 26090271 PMC4469201

[94] Gómez-Conde I Caetano SS Tadokoro CE Olivieri DN. Stabilizing 3D *in vivo* intravital microscopy images with an iteratively refined soft-tissue model for immunology experiments. Comput Biol Med. (2015) 64:246–60. 10.1016/j.compbiomed.2015.07.001 26232672

[95] Soulet D Paré ACoste J Lacroix S. Automated filtering of intrinsic movement artifacts during two-photon intravital microscopy. PLoS ONE. (2013) 8:e53942. 10.1371/journal.pone.0053942 23326545 PMC3543396

[96] Kavanagh DPJ Gallagher MT Kalia N. Tify: a quality-based frame selection tool for improving the output of unstable biomedical imaging. PLoS ONE. (2019) 14:e0213162. 10.1371/journal.pone.0213162 30856207 PMC6411139

